# Human resources and patient rights during COVID-19 pandemic in Iran

**DOI:** 10.18502/jmehm.v13i10.4384

**Published:** 2020-09-16

**Authors:** Seyedhassan Adeli, Morteza Heidari, Akram Heidari

**Affiliations:** 1 *Associate Professor, Spiritual Health Research Center, Qom University of Medical Sciences, Qom, Iran.*; 2 *Researcher, Spiritual Health Research Center, Qom University of Medical Sciences, Qom, Iran. *; 3 *Professor, Spiritual Health Research Center, Qom University of Medical Sciences, Qom, Iran.*

On February 19, 2020, first cases of COVID-19 positive were reported in Iran. When COVID-19 declared pandemic by the World Health Organization (WHO) in March 2020, Iran was among the most affected countries (1). Iran was ranked 11th among pandemic-affected countries with more than 328,844 laboratory-confirmed cases as of August 11, 2020 (2). The rapid spread of the pandemic disease and not knowing about COVID-19-affected areas necessitated making immediate decisions and adopting appropriate control measures. The burden of the pandemic, added to that of the common diseases, imposed additional pressures on hospitals and healthcare centers, with various consequences such as patient’s rights disregard. COVID-19 patients as well as other inpatients during the pandemic deserved their legitimate rights of being treated with the highest possible care and attention. In patient's rights charter (3), various aspects of patient's rights include providing appropriate healthcare service, necessary information, freedom of choice in therapeutic services, and ethical considerations (e.g., privacy and confidentiality). Fulfilling these rights was challenged by shortcomings in hospital capacity, equipment, financial resources, and even necessary knowledge regarding effective treatment. Inadequacy and lack of human resources (4) was a major challenge because healthcare staff count could not be increased to comply with the continuously increasing patient count (4). 

Moreover, loss of staff due to COVID-19 was a serious challenge; in Iran, according to statistics, more than 10,000 medical and healthcare staff were infected and more than 100 deceased as of May 20, 2020 (5). Furthermore, absence from the workplace by medical and healthcare staffs due to the infection risk reduced the capacity of human resources in hospitals and health care centers (6).The healthcare providers and hospital administrators as well as staff, called *Iranian Health Advocates* during COVID-19, were aware of their responsibilities regarding patient’s rights and were committed to doing their best, even if external expectations could not be fully met due to the pandemic.

COVID-19 patients occupied almost the entire capacity of public and private hospitals. The necessity for isolating patients and the large number of patients in need of care and treatment have exacerbated the exhaustion of the nursing staff. To meet the critical need for additional human resources and to improve the situation of human resources, three approaches were adopted: (*i*) Temporary transfer of medical specialists and nursing staff from other divisions of the healthcare system, including educational and research medical centers; (*ii*) Call for retired or non-working nurses and other healthcare specialists to join the healthcare system; and , (*iii*) Recruiting non-specialist volunteers.

To provide non-specialized human resources, use of volunteer force has often been a valuable solution in crisis management (7). In Qom, a city in center of Iran, due to the rapid COVID-19 spread, almost all hospitals were engaged in treating patients. Qom University of Medical Sciences and Health Services, as the main healthcare responsible party for the under-supervision population, as well as the Emergency Operations Center (EOC) issued calls for volunteers through social media. Hundreds of people filled up application forms and enrolled to assist the healthcare staff. The volunteers, most of whom were educated and engaged in non-medical occupations, helped the healthcare system during the difficult days of the epidemic, despite the panic caused by the unknown and insidious virus. After initial screenings, to avoid unpredictable and unintended consequences of utilizing volunteers, empowerment procedures were required for those who volunteered with a sense of benevolence. To ensure fulfilling patient's rights, the volunteers needed training before entering hospitals and healthcare center so that they could perform their expected duties. Therefore, intensive training courses were presented online to the volunteers, and after completing the courses and subsequent exams, ID cards were issued as their permits to enter hospitals and healthcare centers. This training process was necessary for the volunteers in order to acquire basic skills, to activate those skills in establishing effective communication with healthcare staff, and to familiarize them with the workplace climate. Various subjects of the training courses ranged from medical terms to self-care and effective communication to counseling skills. Apart from volunteers who provided logistic support and were not in contact with the patients, 325 volunteers, including 104 females (32%) and 221 males (68%), passed the exams and received ID cards to enter the hospitals as caregivers. Managing of the volunteer caregivers was implemented through a coordinating body consisting of the EOC's public participation committee and the agents in charge of coordination, control, and supervision of the volunteers' activities in the hospitals.

A significant number of the volunteers were Muslim clergies and Islamic seminarian. The volunteers helped fulfill all the basic needs of the patients, especially health care and treatments; however, the volunteer clergies provided the patients with spiritual support. Like chaplain supportive services addressing counselling and psycho-spiritual needs of the medical staff and patients (8), Muslim clergies or Islamic seminarian were more than just volunteers during the pandemic. By incorporating spiritual support into physical care and being in line with patients' religious beliefs, they impact their recovery and well-being both in body and soul. These volunteers responsibly participated in end-of-life care for the end-stage patients, and the subsequent rituals, including burial and spiritual support for families of the deceased, as well as participated in providing support for families of other patients and hospital-isolated medical and healthcare staff. A source of relief for patients, medical and healthcare staff, and their families, these volunteers took on responsibilities that those in charge avoided because of the potential risks of COVID-19. Fulfilling different needs of patients, including their spiritual needs and psychological well-being of their families, was a serious requirement of ensuring the observance of patient’s rights. 

In short, adhering to the patient's rights according to Iranian charter of patient’s right is necessary in all circumstances including COVID-19. During the pandemic, human resource inadequacy was compensated through temporary transferring of medical and healthcare staff from other divisions of healthcare system, recruiting retired or non-working nurses and healthcare specialists, and using volunteer caregivers. During the COVID-19 pandemic, these compensation approaches were effective in fulfilling patient’s rights and avoiding the probable harms of insufficient human resources.

## Notes:

**Citation to this article:**

Adeli SH, Heidari M, Heidari A. Human resources and patient rights during COVID-19 pandemic in Iran. J Med Ethics Hist Med. 2020; 13: 10.
